# Treatment outcomes and risk factors of extra-pulmonary tuberculosis in patients with co-morbidities

**DOI:** 10.1186/s12879-019-4312-9

**Published:** 2019-08-05

**Authors:** Amer Hayat Khan, Syed Azhar Syed Sulaiman, Madeeha Laghari, Mohamed Azmi Hassali, Abdul Razak Muttalif, Zohra Bhatti, Long Chiau Ming, Bandeh Ali Talpur

**Affiliations:** 1 Department of Clinical Pharmacy, School of Pharmaceutical Sciences, Universiti Sains Malaysia, 11800 Gelugor, Penang Malaysia; 2 Faculty of Pharmaceutical Sciences, UCSI University, Kuala Lumpur, Malaysia; 3 Discipline of Social and Administrative Pharmacy, School of Pharmaceutical Sciences, Universiti Sains Malaysia, 11800 Gelugor, Penang Malaysia; 4 Department of Respiratory Medicine, Penang General Hospital, George Town, Penang Malaysia; 5 School of Pharmacy, KPJ Healthcare University College, Nilai, Malaysia; 6 Pharmacy, School of Medicine, University of Tasmania, Hobart, Tasmania Australia; 7 School of Computer Science and Statistics, Trinity College Dublin, Dublin, Ireland

**Keywords:** Extra-pulmonary TB, Co-morbidities, Risk factors, Treatment outcomes

## Abstract

**Background:**

Extra-pulmonary tuberculosis (EPTB) represents about 14% of all cases of tuberculosis (TB) in Malaysia. The aims of the study include evaluation of socio-demographic factors, clinical manifestations, co-morbidities among patients with EPTB and their treatment outcomes.

**Methods:**

A retrospective study was conducted to recognize the epidemiology facts of EPTB. Individual data for EPTB patients were collected from TB registers, laboratory TB registers, treatment cards and TB medical personal files into a standardized study questionnaire. Crude (COR) and adjusted odds ratios (AOR) and 95% confidence intervals (CI) were determined to assess the risk factors for EPTB and unsuccessful treatment outcomes.

**Results:**

There were 1222 EPTB patients presenting 13.1% of all TB cases during 2006–2008. Pleural effusion and lymph node TB were the most frequent types and accounted for 45.1% of all EPTB cases among study participants. Treatment success rate was 67.6%. The best treatment completion rates were found in children ≤15 years (0.478 [0.231–1.028]; *p* = 0.05). On multivariate analysis, age group 56–65 years (1.658 [1.157–2.376]; *p* = 0.006), relapse cases (7.078 [1.585–31.613]; *p* = 0.010), EPTB-DM (1.773 [1.165–2.698]; *p* = 0.008), patients with no formal (2.266 [1.254–4.095]; *p* = 0.001) and secondary level of education (1.889 [1.085–3.288]; *p* = 0.025) were recorded as statistically positive significant risk factors for unsuccessful treatment outcomes. Patients at the risk of EPTB were more likely to be females (1.524 [1.311–1.746]; *p* <  0.001), Malays (1.251 [1.056–1.482]; *p* = 0.010) and Indians (1.450 [1.142–1.842]; *p* = 0.002), TB-HIV (3.215 [2.347–4.405]; *p* <  0.001), EPDM-HIV (4.361 [1.657–11.474]; *p* = 0.003), EPTB-HIV-HEP (4.083 [2.785–5.987]; *p* <  0.001), those living in urban areas (1.272 [1.109–1.459]; *p* = 0.001) and no formal education (1.361 [1.018–1.820]; *p* = 0.037).

**Conclusion:**

The findings of this study extend the knowledge of EPTB epidemiology and highlight the need for improved EPTB detection in Malaysia, especially in subpopulations with high risk for EPTB and unsuccessful treatment outcomes.

## Background

TB is the ninth foremost reason of death worldwide and the leading cause from a single infectious agent, ranking above HIV/AIDS [1]. Overall, a relatively small proportion (5–15%) of the people infected with *Mycobacterium tuberculosis* (MTB) will develop TB disease during their lifetime. However, the probability of developing TB disease is much higher among people infected with HIV, and also higher among people affected by risk factors such as under-nutrition, diabetes, smoking and alcohol consumption [1].

Pulmonary TB (PTB), the most common type of TB, has the great epidemiological significance due to its extremely contagious nature [2]. The proportion of patients with extra-pulmonary TB (EPTB) relative to those with PTB varies among countries and depends on associated diseases, geographical, social, ethnic and economic parameters [3, 4]. EPTB is defined according to WHO classification criteria as an infection by MTB which affects tissues and organs outside the pulmonary parenchyma. It represents between 20 and 25% of all TB cases [5].

In the early 1940s and 1950s, TB was graded as the main reason of death in Malaysia. Realizing its seriousness, the Malaysian government launched its National TB Control Program (NTP) in 1961 [6]. With regards to the type of TB, of 25,739 reported TB cases in Malaysia during 2016, 22,135 (86%) were PTB cases while 3604 (14%) were EPTB [1]. The most common forms of EPTB seen in Malaysia are TB lymphadenitis, bone/joint TB and miliary TB [6]. From 1990 to 2016, the number of TB-HIV co-infection reported nationwide has increased from 6 to 3396 cases [7]. EPTB involvement tends to increase in frequency if the immune system is compromised [2]. Diabetes Mellitus (DM) has been identified as a risk factor for TB [8–10]. Although immune deficiency also occurs with diabetes, but little is known about the epidemiological or clinical relationship between diabetes and EPTB. However, we hypothesized that infectious and immune-compromised conditions increased frequency and severity of EPTB. The current study was conducted with the aim to evaluate the clinical characteristics and treatment outcomes of patients with EPTB. Furthermore, we were interested to identify the risk factors of EPTB on socio-demographic, co-morbidities and clinical basis.

## Methods

### Study design and data collection

A retrospective study was conducted in four states of Malaysia from 2006 to 2008. Data on socio-demographic, clinical, histopathological, microbiological and other laboratory variables of EPTB cases were collected from TB registers, treatment cards and TB medical personal files using standard data collection tool. Patients in whom the site of infection was confined to lungs were considered as PTB while infection extended to other organs or tissues outside lungs were considered as EPTB. Patients who had both PTB and EPTB involvements were excluded from the analysis of EPTB based on WHO sample selection policy [11]. The sites of infection were as lymph nodes, gastrointestinal system, spinal, meningitis pleural effusion, miliary and bones/joints. All other sites of infection were considered as part of a seventh group identified as “other rare forms” (including urogenital, eyes, ear, breast etc.) for convenient statistical analysis the diagnosis of EPTB was done following Clinical Practice Guidelines [12], which is consistent with the WHO’s diagnostic criteria [13]. Treatment success refers to the patients who were cured and have completed TB treatment. Completed TB treatment was defined as any patient who had completed a TB regime based on the Clinical Practice Guidelines created by Malaysia Ministry of Health [12].

### Study location

The study was carried out in selected hospitals and prisons located in four states of Malaysia (Penang, Sabah, Sarawak, and Selangor). The states of Penang, Sabah and Sarawak were selected for the present study based on previous literature report of TB burden. The prevalence of TB in Malaysia was highest in Sabah followed by Sarawak and Penang [6]. The state of Selangor was also considered for the present study as it has two big prisons at national level in Malaysia.

### Data analysis

The whole data collection form was assigned a serial number to ensure the traceability. Coding of the responses was carried out and the data was entered into the computer and analysed by using statistical package for SPSS for Windows version 16.0 (SPSS, Inc., Chicago, IL, USA). Logistic regression model was used to analyze the predictors for EPTB and unsuccessful treatment outcomes. However, *p* value was used to calculate significance of of co-morbidities among different variables. Factors found significant in univariate analysis, were finally included in multivariate logistic regression to estimate the odds ratios (ORs) with their 95% confidence intervals (CIs). A *p* value < 0.05 was considered statistically significant.

## Results

### Proportion, socio-demographic and baseline clinical characteristics

Proportion of EPTB registered in four states of Malaysia during study time period is shown in Table 1. Patients with EPTB constituted about 13.1% of all TB cases, with higher prevalence in Selangor than other three states. Socio-demographic and clinical characteristics of patients are shown in Table 2. The study cohort included 778 (63.7%) males and 444 (36.3%) females. Of the 1222 cases included in the present study, age group 26–35 years constituted the highest cases (24.2%) and least numbers were recorded for ≤15 years old (4.5%). In terms of residence, higher proportions of EPTB were observed in the urban areas (67%). The results showed that there was a significant difference (*p* <  0.001) in the incidence of EPTB among different races. The Malays seemed to be highly affected with a number of 389 (31.8%) cases followed by the Chinese (23.9%).

**Table 1:** Proportion of PTB and EPTB registered in four states of Malaysia during 2006–2008

States	PTB *n* (%)	EPTB *n* (%)	Total
Penang	1285 (84.8)	230 (15.2)	1515
Sabah	3803 (88.3)	504 (11.7)	4307
Sarawak	1722 (91.5)	160 (8.5)	1882
Selangor	1303 (79.9)	328 (20.1)	1631
Total	8113	1222	9335

*PTB* Pulmonary TB, *EPTB* Extra- Pulmonary TB

**Table 2:** Socio-demographic and clinical characteristics of EPTB patients (*n* = 1222)

Variables	Total *n* = 1222 (%)	Lymph node *n* = 324 (%)	Pleural effusion *n* = 227 (%)	Meningitis *n* = 122 (%)	Miliary *n* = 113 (%)	Bones *n* = 116 (%)	Gastro-intestinal *n* = 105 (%)	Spinal *n* = 93 (%)	Others *n* = 122 (%)
Gender									
Male	778(63.7)	214 (27.5)	155 (19.9)	75 (9.6)	83 (10.7)	72 (9.3)	55 (7.1)	61 (7.8)	63 (8.1)
Female	444 (36.3)	110 (24.8)	72 (16.2)	47 (10.6)	30 (6.8)	44 (9.9)	50 (11.3)	32 (7.2)	59 (13.3)
Age (years)									
≤ 15	55 (4.5)	14 (25.5)	7 (12.7)	7 (12.7)	2 (3.6)	0	11 (20)	4 (7.3)	10 (18.2)
16–25	177 (14.5)	50 (28.2)	28 (15.8)	13 (7.3)	13 (7.3)	21 (11.9)	26 (14.7)	4 (2.3)	22 (12.4)
26–35	296 (24.2)	118 (39.9)	33 (11.1)	28 (9.5)	30 (10.1)	34 (11.5)	17 (5.7)	10 (3.4)	26 (8.8)
36–45	224 (18.3)	54 (24.1)	42 (18.8)	25 (11.2)	21 (9.4)	23 (10.3)	16 (7.1)	28 (12.5)	15 (6.7)
46–55	233 (19.1)	46 (19.7)	47 (20.2)	20 (8.6)	23 (9.9)	23 (9.9)	24 (10.3)	27 (11.6)	23 (9.9)
56–65	170 (13.9)	36 (21.2)	46 (27.1)	23 (13.5)	15 (8.8)	11 (6.5)	9 (5.3)	14 (8.2)	16 (9.4)
≥ 66	67 (5.5)	6 (9)	24 (35.8)	6 (9)	9 (13.4)	4 (6)	2 (3)	6 (9)	10 (14.9)
Race									
Malay	389(31.8)	132 (33.9)	62 (15.9)	26 (6.7)	34 (8.7)	39 (10)	31 (8)	31 ((8)	34 (8.7)
Chinese	292 (23.9)	78 (26.7)	61 (20.9)	26 (8.9)	26 (8.9)	29 (9.9)	24 (8.2)	23 (7.9)	25 (8.6)
Indian	128 (10.5)	34 (26.6)	30 (23.4)	6 (4.7)	16 (12.5)	10 (7.8)	12 (9.4)	10 (7.8)	10 (7.8)
Immigrants Indonesian	63 (5.2)	13 (20.6)	16 (25.4)	7 (11.1)	7 (11.1)	7 (11.1)	5 (7.9)	4 (6.3)	4 (6.3)
Immigrants Philippines	45 (3.7)	4 (8.9)	6 (13.3)	11 (24.4)	2 (4.4)	2 (4.4)	8 (17.8)	3 (6.7)	9 (14.3)
Sarawakian	87 (7.1)	15 (17.2)	25 (28.7)	3 (3.4)	2 (2.3)	12 (13.8)	2 (2.3)	7 (8)	21 (24)
Sabahian	189 (15.5)	36 (19)	18 (9.5)	42 (22.2)	26 (13.8)	16 (8.5)	21 (11.1)	13 (6.9)	17 (9)
Others	29 (2.4)	12 (41.4)	9 (31)	1 (3.4)	0	1 (3.4)	2 (6.9)	2 (6.9)	2 (6.9)
Residence									
Urban	819 (67)	194 (23.7)	177 (21.6)	77 (9.4)	66 (8.1)	81 (10)	75 (9.2)	56 (6.8)	93 (11.4)
Rural	403 (33)	130 (32.3)	50 (12.4)	45 (11.2)	47 (11.7)	35 (8.7)	30 (7.4)	37 (9.2)	29 (7.2)
Patient category									
Unknown	23 (1.9)	5 (21.7)	7 (30.4)	0	4 (17.4)	3 (13)	0	1 (4.3)	3 (13)
New	1131 (92.6)	304 (26.9)	206 (18.2)	115 (10.2)	103 (9.1)	110 (9.7)	95 (8.4)	86 (7.6)	112 (9.9)
Relapse	68 (5.6)	15 (22.1)	14 (20.6)	7 (10.3)	6 (8.8)	3 (4.4)	10 (14.7)	6 (8.8)	7 (10.3)
Co-morbidity									
Only TB	697 (57)	165 (23.7)	111 (15.9)	70 (10)	75 (10.8)	74 (10.6)	68 (9.8)	50 (7.2)	84 (12.1)
TB-DM	180 (14.7)	40 (22.2)	61 (33.9)	16 (8.9)	7 (3.9)	10 (5.6)	17 (9.4)	13 (7.2)	16 (8.9)
TB-HIV	188 (15.4)	70 (37.2)	22 (11.7)	20 (10.6)	18 (9.6)	23 (12.2)	11 (5.9)	9 (4.8)	15 (8)
TB-Hep	60 (4.9)	9 (15)	16 (26.7)	6 (10)	4 (4.7)	3 (5)	3 (5)	15 (25)	4 (4.7)
TB-DM-HIV	7 (0.6)	3 (42.9)	2 (28.6)	1 (14.3)	0	0	0	0	1 (14.3)
TB-DM-Hep	11 (0.9)	1 (9.1)	2 (18.2)	2 (18.2)	1 (9.1)	0	4 (36.4)	1 (9.1)	0
TB-HIV-Hep	79 (6.5)	36 (45.6)	13 (16.5)	7 (8.9)	8 (10.1)	6 (7.6)	2 (2.5)	5 (6.3)0	2 (2.5)
Smoking habit									
Ex-smoker	166 (13.6)	28 (16.9)	33 (19.9)	12 (7.2)	24 (14.5)	12 (7.2)	27 (16.3)	7 (4.2)	23 (13.9)
No	647 (52.9)	180 (27.8)	114 (17.6)	62 (9.6)	57 (8.8)	56 (8.7)	58 (7)	50 (7.7)	70 (10.8)
Yes	409 (33.5)	116 (28.4)	80 (19.6)	48 (11.7)	32 (7.8)	48 (11.7)	20 (4.9)	36 (8.8)	29 (7.1)
Drinking Habit									
Unknown	12 (1)	2 (16.7)	2 (16.7)	2 (16.7)	0	0	1 (8.3)	1 (8.3)	4 (33.3)
No	1094 (89.5)	291 (26.6)	207 (18.9)	110 (10.1)	96 (8.8)	105 (9.6)	97 (8.9)	78 (7.1)	110 (10.1)
Yes	116 (9.5)	31 (26.7)	18 (15.5)	10 (8.6)	17 (14.7)	11 (9.5)	7 (6)	14 (12.1)	8 (6.9)
IVDU^a^									
Unknown	23 (1.9)	7 (30.4)	7 (30.4)	1 (4.3)	3 (13)	1 (4.3)	2 (8.7)	1 (4.3)	1 (4.3)
No	1113 (91)	301 (27)	205 (18.4)	105 (9.4)	100 (9)	111 (10)	96 (8.6)	86 (7.8)	109 (9.8)
Yes	86 (7.1)	16 (18.6)	15 (17.4)	16 (18.6)	10 (11.6)	4 (4.7)	7 (8.1)	6 (7)	12 (14)
Marital status									
Unknown	22 (1.8)	4 (18.2)	6 (27.3)	2 (9.1)	5 (22.7)	2 (9.1)	0	0	3 (13.6)
Married	539 (44.1)	99 (26.9)	72 (19.6)	35 (9.5)	46 (12.5)	32 (8.7)	35 (9.5)	17 (4.6)	32 (8.7)
Unmarried	832 (68.1)	221 (26.6)	149 (17.9)	85 (10.2)	62 (7.5)	82 (9.9)	70 (8.4)	76 (9.1)	87 (10.5)
Education									
Unknown	876 (71.7)	224 (25.6)	162 (18.5)	97 (11.1)	84 (9.6)	78 (8.9)	80 (9.1)	63 (7.2)	88 (10)
Primary	111 (9.1)	40 (36)	13 (11.7)	7 (6.3)	9 (8.1)	13 (11.7)	8 (7.2)	7 (6.3)	14 (12.6)
Secondary	84 (6.9)	17 (20.2)	22 (26.2)	8 (9.5)	6 (7.1)	8 (9.5)	10 (11.9)	5 (6)	8 (9.5)
College	42 (3.4)	23 (54.8)	7 (16.7)	0	1 (2.4)	1 (2.4)	2 (4.8)	5 (11.9)	3 (7.1)
University	8 (0.7)	1 (12.5)	3 (37.5)	0	1 (12.5)	0	0	1 (12.5)	2 (25)
Diploma	20 (1.6)	5 (25	1 (5)	0	0	10 (50)	1 (5)	3 (15)	0
No formal education	81 (6.6)	14 (17.3)	19 (23.5)	10 (12.3)	12 (14.8)	6 (7.4)	4 (4.9)	9 (11.1)	7 (8.6)
Employment status									
Unknown	188 (15.4)	54 (28.7)	28 (14.9)	9 (4.8)	22 (11.7)	15 (8)	16 (8.5)	33 (17.6)	11 (5.9)
Employed	342 (28)	104 (30.4)	67 (19.6)	30 (8.8)	25 (7.3)	42 (12.3)	22 (6.4)	26 (7.6)	26 (7.6)
Unemployed	692 (56.6)	166 (24)	132 (19.1)	83 (12)	66 (9.5)	59 (8.5)	67 (9.7)	34 (4.9)	85 (12.3)

*TB-DM* Co-infection of TB and Diabetes Mellitus, *TB-HIV* Co-infection of TB and HIV, *TB-Hep* Co-infection of TB and Hepatitis, *TB-DM-HIV* Co-infection of TB with Diabetes Mellitus and HIV, *TB-DM-Hep* Co-infection of TB with Diabetes Mellitus and Hepatitis, *TB-HIV-Hep* Co-infection of TB with HIV and Hepatitis

^a^Intravenous Drug Users

Around, 330 (27%) of cases had acid fast bacilli smear positive and 687 (56.2%) culture positive. However, 360 patients (29.5%) were diagnosed via pathology alone and 43 (3.5%) were confirmed on polymerase chain reaction (PCR). Among the culture confirmed EPTB cases, 1.5% of patients were resistant to single first line drugs. On baseline 4 patients were recorded resistant to isoniazid and 4 for streptomycin whereas 1 each for rifampicin and ethambutol. Of HIV co-infection, 139 patients were receiving Highly Active Antiretroviral Therapy (HAART). For EPTB patients with DM, 152 were getting oral hypoglycemics, 24 patients were on insulin whereas 6 had oral hypoglycemic agents plus insulin.

### Frequency distribution of EPTB

Of 9335 all TB cases registered during the study time period, 1222 had EPTB. Lymph node 324 (26.5%) and Pleural effusion 227 (18.6%) TB were the most frequent types of all EPTB cases among study participants (Table 2). The proportions of different types of EPTB varied with statistically significant difference observed among gender (*p* = 0.03), age groups (*p* <  0.001), different ethnic groups (*p* <  0.001), co-morbidities (*p* <  0.001), and smokers (*p* <  0.001) (Table 2).

### EPTB and co-morbidities

Out of 1222 EPTB patients, 525 (43%) were recorded with co-morbidities. Chi-square analysis of categorical variables of the study participants showed a significant difference in males and females, distribution of age, races, residence, patient categories, marital status, education and employment between EPTB and comorbidities groups (Table 3). Among the co-morbidities, HIV and DM contributed to the highest cases and almost at the equal rate. Overall, higher proportions of all comorbidities were seen among males than females and patients aged 35 years or older. Numbers of EPTB with diabetes mellitus (EPTB-DM), EPTB with human immunodeficiency virus (EPTB-HIV) and EPTB and hepatitis (EPTB-HEP) cases were frequently seen among 35–55 years age whereas the co-morbidities became more complex with increasing age. Patients from the rural areas were significantly had increased proportions of HIV related comorbidities. Moreover, patients with CD4+ lymphocyte cell counts < 100 had 37.3% lymph node TB, 18.6% cases of miliary and pleural TB each. We further confirmed that of total deaths among known CD4+ lymphocyte count, 55% occurred in patients with < 100 counts. Other diseases which patients already had at the baseline include hypertension (3%), ischemic heart disease (1%), renal failure (2%), lung carcinoma (0.2%), lung fibrosis (0.2%), liver cirrhosis (0.2%), hypertension and COPD (0.2%), ischemic heart disease+ renal failure+ hypertension (0.4%) (Table 3).

**Table 3:** Distribution and frequency of different types of co-morbidities among patients with EPTB (*n* = 1222)

Variables	Total 1222 (%)	TB only *n* = 697 (%)	TB-DM *n* = 180 (%)	TB-HIV *n* = 188 (%)	TB-Hepatitis *n* = 60 (%)	TB-DM-HIV *n* = 7 (%)	TB-DM-Hepatitis *n* = 11 (%)	TB-HIV-Hepatitis *n* = 79 (%)	*P*-value
Gender									
Male	778(63.7)	381 (54.7)	93 (51.7)	178 (94.7)	33 (55)	7 (100)	10 (90.1)	76 (96.2)	< 0.001
Female	444 (36.3)	316 (45.3)	87 (48.3)	10 (5.3)	27 (45)	0	1 (9.9)	3 (3.8)	
Age (years)									
≤ 15	55 (4.5)	47 (6.7)	3 (1.7)	1 (0.5)	2 (3.3)	1 (14.3)	0	1 (1.3)	
16–25	177 (14.5)	136 (19.5)	11 (6.1)	13 (6.9)	8 (13.3)	1 (14.3)	2 (18.2)	6 (7.6)	
26–35	296 (24.2)	169 (24.2)	30 (16.7)	66 (35)	9 (15)	1 (14.3)	2 (18.2)	19 (24.1)	< 0.001
36–45	224 (18.3)	103 (14.8)	22 (12.2)	52 (27.7)	15 (25)	1 (14.3)	0	31 (39.2)	
46–55	233 (19.1)	114 (16.4)	54 (30)	36 (19.1)	14 (23.3)	0	0	15 (19)	
56–65	170 (13.9)	81 (11.6)	51 (28.3)	16 (8.5)	7 (11.7)	3 (42.9)	6 (54.5)	6 (7.6)	
≥ 66	67 (5.5)	47 (6.7)	9 (5)	4 (2.1)	5 (8.3)	0	1 (9.1)	1 (1.3)	
Race									
Malay	389(31.8)	183 (26.3)	54 (30)	89 (47.3)	22 (36.7)	2 (28.6)	4 (36.4)	35 (44.3)	
Chinese	292 (23.9)	155 (22.2)	43 (23.9)	52 (27.7)	19 (31.7)	2 (28.6)	2 (18.2)	19 (24.1)	
Indian	128 (10.5)	66 (9.5)	21 (11.7)	21 (11.2)	6 (10)	2 (28.6)	2 (18.2)	10 (12.7)	
Immigrants Indonesian	63 (5.2)	40 (5.7)	12 (6.7)	5 (2.7)	2 (3.3)	0	0	4 (5.1)	< 0.001
Immigrants Philippines	45 (3.7)	30 (4.3)	12 (6.7)	1 (0.5)	0	0	1 (9.1)	1 (1.3)	
Sarawakian	87 (7.1)	58 (8.3)	15 (8.3)	7 (3.7)	3 (5)	0	0	4 (5.1)	
Sabahian	189 (15.5)	149 (21.4)	22 (12.2)	5 (2.7)	7 (11.7)	1 (14.3)	2 (18.2)	3 (3.8)	
Others	29 (2.4)	16 (2.3)	1 (0.6)	8 (4.3)	1 (1.7)	0	0	3 (3.8)	
Patient category									
Unknown	23 (1.9)	9 (1.3)	1 (0.6)	11 (5.9)	0	0	0	2 (2.5)	< 0.001
New	1131 (92.6)	652 (93.5)	169 (93.8)	166 (88.2)	57 (95)	7 (100)	6 (54.5)	74 (93.7)	
Relapse	68 (5.6)	36 (5.2)	10 (5.6)	11 (5.9)	3 (5)	0	5 (45.5)	3 (3.8)	
Residency									
Urban	819 (67)	494 (70.9)	149 (82.8)	88 (46.8)	46 (76.7)	1 (14.3)	10 (90.1)	31 (39.2)	< 0.001
Rural	403 (33)	203 (29.1)	31 (17.2)	100 (53.2)	14 (23.3)	6 (85.7)	1 (9.1)	48 (60.8)	
Smoking habit									
Ex-smoker	166 (13.6)	105 (15.1)	38 (21.1)	8 (4.3)	11 (18.3)	0	1 (9.1)	3 (3.8)	< 0.001
No	647 (52.9)	402 (57.7)	91 (50.6)	85 (45.2)	28 (46.7)	1 (14.3)	5 (45.5)	35 (44.3)	
Yes	409 (33.5)	190 (27.3)	51 (28.3)	95 (50.5)	21 (35)	6 (85.7)	5 (45.5)	41 (51.9)	
Drinking Habit									
Unknown	12 (1)	5 (0.7)	2 (1.1)	2 (1.1)	0	0	0	3 (3.8)	< 0.001
No	1094 (89.5)	660 (94.7)	176 (97.8)	127 (67.6)	56 (93.3)	5 (71.4)	10 (90.1)	60 (75.9)	
Yes	116 (9.5)	32 (4.6)	2 (1.1)	59 (31.4)	4 (6.7)	2 (28.6)	1 (9.1)	16 (20.3)	
IVDU^a^									
Unknown	23 (1.9)	7 (1)	0	2 (1.1)	1 (1.7)	1 (14.3)	0	12 (15.2)	< 0.001
No	1113 (91)	659 (94.5)	177 (98.3)	152 (80.9)	53 (88.3)	4 (57.1)	10 (90.1)	58 (73.4)	
Yes	86 (7.1)	31 (4.4)	3 (1.7)	34 (18.1)	6 (10)	2 (28.6)	1 (9.1)	9 (11.4)	
Marital status									
Unknown	22 (1.8)	6 (0.9)	1 (0.6)	12 (6.4)	0	0	0	3 (3.8)	< 0.001
Married	539 (44.1)	224 (32.1)	45 (25)	51 (27.1)	18 (30)	1 (14.3)	1 (9.1)	28 (35.5)	
Unmarried	832 (68.1)	467 (67)	134 (74.4)	125 (66.5)	42 (70)	6 (85.7)	10 (90.1)	48 (60.8)	
Education									
Unknown	876 (71.7)	512 (73.5)	119 (66.1)	121 (64.4)	46 (76.7)	4 (57.1)	8 (72.7)	65 (82.3)	
Primary	111 (9.1)	69 (9.9)	15 (8.3)	17 (9)	3 (5)	1 (14.3)	1 (9.1)	6 (7.6)	
Secondary	84 (6.9)	52 (7.5)	23 (12.8)	4 (2.1)	3 (5)	1 (14.3)	1 (9.1)	0	
College	42 (3.4)	20 (2.9)	10 (5.6)	8 (4.3)	3 (5)	0	0	1 (1.3)	< 0.001
University	8 (0.7)	5 (0.7)	0	3 (1.6)	0	0	0	0	
Diploma	20 (1.6)	7 (1)	2 (1.1)	9 (4.8)	0	0	0	2 (2.5)	
No formal education	81 (6.6)	32 (4.6)	11 (6.1)	26 (13.8)	5 (8.3)	1 (14.3)	1 (9.1)	5 (6.3)	
Employment status									
Unknown	188 (15.4)	76 (10.9)	26 (14.4)	67 (35.6)	4 (6.7)	0	1 (9.1)	14 (17.7)	< 0.001
Employed	342 (28)	200 (28.7)	63 (35)	44 (23.4)	19 (31.7)	4 (57.1)	4 (36.4)	12 (15.2)	
Unemployed	692 (56.6)	421 (60.4)	91 (50.6)	77 (41)	37 (61.7)	3 (42.9)	6 (54.5)	53 (67.1)	

*TB-DM* Co-infection of TB and Diabetes Mellitus, *TB-HIV* Co-infection of TB and HIV, *TB-Hepatitis* Co-infection of TB and Hepatitis, *TB-DM-HIV* Co-infection of TB with Diabetes Mellitus and HIV, *TB-DM-Hepatitis* Co-infection of TB with Diabetes Mellitus and Hepatitis, *TB-HIV-Hepatitis* Co-infection of TB with HIV and Hepatitis

^a^Intravenous Drug Users

### Treatment outcomes

Around, 67.6% (826/1222) patients successfully completed treatment. Treatment outcomes in relation to socio-demographic characteristics and co-morbidities are shown in Table 4. There were no statistically significant differences seen with regard to treatment outcomes among males and females, residency, ethnicity, alcohol habit and employment on univariate analysis (Table 4).

**Table 4:** Logistic regression models to determine independent risk factors for unsuccessful treatment outcomes among EPTB patients

Variables	Total 1222	Treatment outcomes		Univariate analysis	*P-* value	Multivariate analysis	*P*-value
	*n* (%)	Successful *n* (%)	Unsuccessful *n* (%)	COR (95% CI)		AOR (95% CI)	
Gender							
Male	778(63.7)	514 (66.1)	264 (33.9)	0.82 (0.64 to 1.06)	0.131	---------------------	-------
Female	444 (36.3)	312 (70.3)	132 (29.7)	1.21 (0.94 to 1.56)			
Age (years)							
≤ 15	55 (4.5)	46 (83.6)	9 (16.4)	0.39 (0.19 to 0.81)	0.012	0.47 (0.23 to 1.02)	0.050
16–25	177 (14.5)	129 (72.9)	48 (27.1)	0.67 (0.48 to 0.99)	0.094	---------------------	-------
26–35	296 (24.2)	203 (68.6)	93 (31.4)	0.94 (0.71 to 1.24)	0.677	---------------------	-------
36–45	224 (18.3)	146 (65.2)	78 (34.8)	1.14 (0.84 to 1.55)	0.393	---------------------	-------
46–55	233 (19.1)	167 (71.7)	66 (28.3)	0.78 (0.57 to 1.08)	0.140	---------------------	-------
56–65	170 (13.9)	90 (52.9)	80 (47.1)	2.07 (1.49 to 2.87)	≤ 0.001	1.65 (1.15 to 2.37)	0.006
≥ 66	67 (5.5)	43 (64.2)	24 (35.8)	1.17 (0.70 to 1.96)	0.539	---------------------	-------
Ethnicity							
Malay	389(31.8)	264 (67.9)	125 (32.1)	0.98 (0.75 to 1.27)	0.889	---------------------	-------
Chinese	292 (23.9)	192 (65.8)	100 (34.2)	1.11 (0.84 to 1.47)	0.441	---------------------	-------
Indian	128 (10.5)	80 (62.5)	48 (37.5)	1.28 (0.88 to 1.88)	0.194	---------------------	-------
Immigrants Indonesian	63 (5.2)	42 (66.7)	21 (33.3)	1.04 (0.61 to 1.79)	0.872	---------------------	-------
Immigrants Philippines	45 (3.7)	31 (68.9)	14 (31.1)	0.94 (0.49 to 1.78)	0.850	---------------------	-------
Sarawakian	87 (7.1)	75 (86.2)	12 (13.8)	0.31 (0.16 to 0.58)	≤ 0.001	0.36 (0.19 to 0.69)	0.003
Sabahian	189 (15.5)	122 (64.6)	67 (35.4)	1.17 (0.84 to 1.62)	0.331	---------------------	-------
Others	29 (2.4)	20 (69)	9 (31)	0.93 (0.42 to 2.07)	0.873	---------------------	-------
Residency							
Urban	819 (67)	551 (67.3)	268 (32.7)	1.04 (0.80 to 1.34)	0.736	---------------------	-------
Rural	403 (33)	275 (68.2)	128 (31.8)	0.95 (0.74 to 1.23)			
Patient category							
Unknown	23 (1.9)	20 (87)	3 (13)	0.30 (0.09 to 1.04)	0.058	---------------------	-------
New	1131 (92.6)	773 (68.3)	358 (31.7)	0.64 (0.41 to 0.99)	0.049	3.27 (0.79 to 13.42)	0.099
Relapse	68 (5.6)	33 (48.5)	35 (51.5)	2.33 (1.42 to 3.80)	0.001	7.07 (1.58 to 31.61)	0.010
Co-morbidity							
Only TB	697 (57)	494 (70.9)	203 (29.1)	0.70 (0.55 to 0.90)	0.005	1.01 (0.73 to 1.38)	0.938
TB-DM	180 (14.7)	98 (54.4)	82 (45.6)	1.94 (1.40 to 2.67)	≤ 0.001	1.77 (1.16 to 2.69)	0.008
TB-HIV	188 (15.4)	129 (15.4)	59 (31.4)	0.94 (0.67 to 1.32)	0.745	---------------------	-------
TB-Hepatitis	60 (4.9)	43 (71.7)	17 (28.3)	0.81 (0.46 to 1.45)	0.490	---------------------	-------
TB-DM-HIV	7 (0.6)	2 (28.6)	5 (71.4)	5.26 (1.01 to 27.27)	0.048	4.13 (0.76 to 22.40)	0.100
TB-DM-Hepatitis	11 (0.9)	3 (27.3)	8 (72.7)	5.69 (1.50 to 21.58)	0.011	3.42 (0.83 to 14.07)	0.087
TB-HIV-Hepatitis	79 (6.5)	56 (70.9)	23 (29.1)	0.90 (0.54 to 1.47)	0.679	----------------------	-------
Smoking habit							
Ex-smoker	166 (13.6)	118 (71.1)	48 (28.9)	0.82 (0.57 to 1.18)	0.291	---------------------	-------
No	647 (52.9)	455 (70.3)	192 (29.7)	0.76 (0.59 to 0.96)	0.026	1.03 (0.69 to 1.54)	0.856
Yes	409 (33.5)	253 (61.9)	156 (38.1)	1.48 (1.15 to 1.91)	0.002	1.34 (0.86 to 2.07)	0.185
Drinking Habit							
Unknown	12 (1)	5 (41.7)	7 (58.3)	2.94 (0.92 to 9.33)	0.067		
No	1094 (89.5)	741 (67.7)	353 (32.3)	0.84 (0.57 to 1.23)	0.379	---------------------	-------
Yes	116 (9.5)	80 (69)	36 (31)	1.05 (0.70 to 1.58)	0.784		
IVDU^a^							
Unknown	23 (1.9)	11 (47.8)	12 (52.2)	2.30 (1.00 to 5.27)	0.048	1.79 (0.68 to 4.73)	0.237
No	1113 (91)	761 (68.4)	352 (31.6)	0.65 (0.44 to 0.98)	0.041	0.91 (0.54 to 1.54)	0.732
Yes	86 (7.1)	54 (62.8)	32 (37.2)	1.32 (0.84 to 2.07)	0.228	----------------------	-------
Marital status							
Unknown	22 (1.8)	17 (77.3)	5 (22.7)	0.77 (0.30 to 1.99)	0.599	----------------------	-------
Married	539 (44.1)	265 (72)	103 (28)	0.75 (0.57 to 0.98)	0.039	1.27 (0.39 to 4.09)	0.681
Unmarried	832 (68.1)	544 (65.4)	288 (34.6)	1.34 (1.03 to 1.74)	0.029	1.29 (0.41 to 4.01)	0.652
Education							
Unknown	876 (71.7)	627 (71.7)	249 (28.5)	0.53 (0.41 to 0.68)	≤ 0.001	0.73 (0.49 to 1.07)	0.112
Primary	111 (9.1)	67 (59.8)	44 (39.2)	1.44 (0.97 to 2.15)	0.069	----------------------	------
Secondary	84 (6.9)	41 (48.8)	43 (51.2)	2.32 (1.48 to 3.62)	≤ 0.001	1.88 (1.08 to 3.28)	0.025
College	42 (3.4)	30 (71.4)	12 (28.6)	0.82 (0.41 to 1.63)	0.582	---------------------	--------
University	8 (0.7)	4 (50)	4 (50)	2.08 (0.52 to 8.39)	0.299	---------------------	--------
Diploma	20 (1.6)	18 (90)	2 (10)	0.22 (0.05 to 0.98)	0.047	0.20 (0.04 to 0.91)	0.038
No formal education	81 (6.6)	38 (46.9)	43 (53.1)	2.51 (1.59 to 3.96)	≤ 0.001	2.26 (1.25 to 4.09)	0.001
Employment status							
Unknown	188 (15.4)	129 (68.6)	59 (31.4)	0.94 (0.67 to 1.31)	0.725	---------------------	--------
Employed	342 (28)	226 (66.1)	116 (33.9)	1.09 (0.83 to 1.42)	0.514		
Unemployed	692 (56.6)	470 (67.9)	222 (32.1)	0.96 (0.75 to 1.22)	0.742		

*TB-DM* Co-infection of TB and Diabetes Mellitus, *TB-HIV* Co-infection of TB and HIV, *TB-Hepatitis* Co-infection of TB and Hepatitis, *TB-DM-HIV* Co-infection of TB with Diabetes Mellitus and HIV, *TB-DM-Hepatitis* Co-infection of TB with Diabetes Mellitus and Hepatitis, *TB-HIV-Hepatitis* Co-infection of TB with HIV and Hepatitis

^a^Intravenous Drug Users

**Fig. 1:**
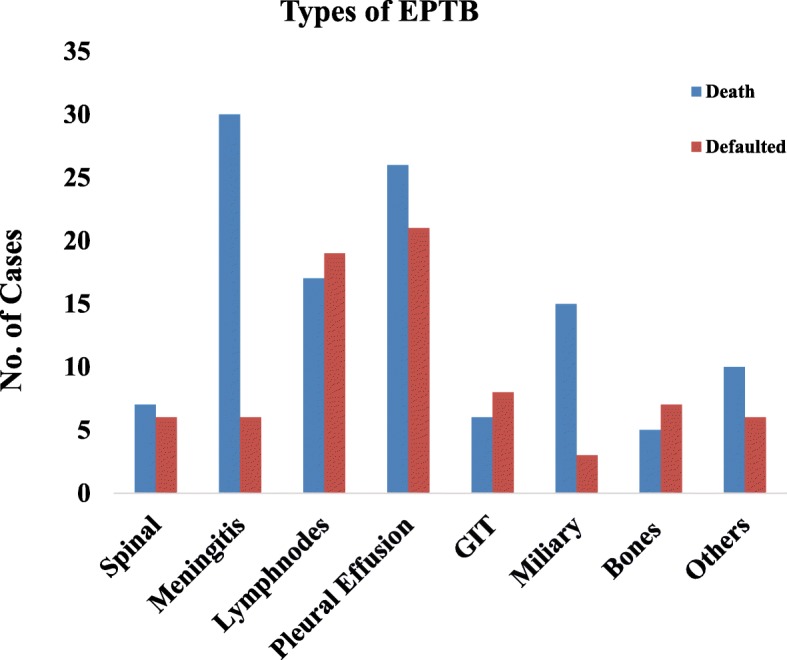
Proportion of default and deaths among different types of EPTB among patients

On multivariate analysis (Table 4), age group 56–65 years (1.658 [1.157–2.376]; *p* = 0.006), relapse cases (7.078 [1.585–31.613]; *p* = 0.010), EPTB-DM (1.773 [1.165–2.698]; *p* = 0.008), patients with no formal (2.266 [1.254–4.095]; *p* = 0.001) and secondary level of education (1.889 [1.085–3.288]; *p* = 0.025) were recorded as statistically positive significant risk factors for unsuccessful treatment outcomes. Comparing the proportion of default and deaths among different types of EPTB, significantly higher were reported in meningitis and miliary TB (Fig. 1).

**Fig. 2:**
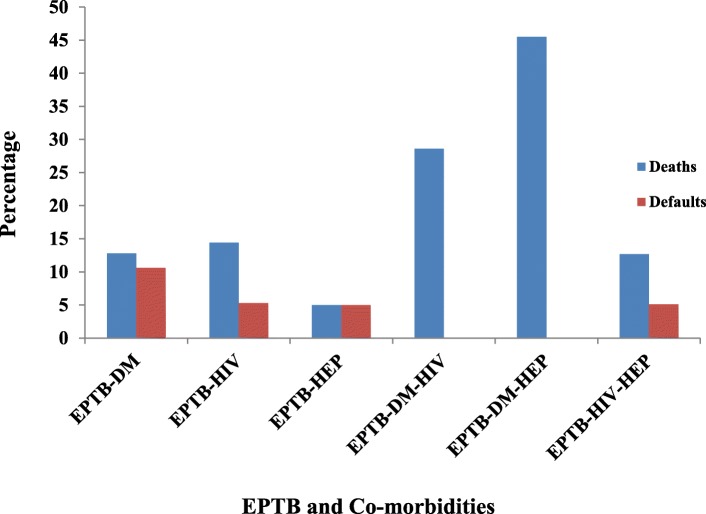
Proportion of default and deaths among patients with EPTB and different co-morbidities

Of 139 patients who were on HAART, 111 (79.9%) successfully completed the treatment while the remaining 28 (20.1%) had unsuccessful treatment outcomes. Nevertheless, statistically significant association was observed between DM and treatment outcomes for EPTB-DM patients. When death and default cases were compared among the different co-morbidities, maximum death cases were observed for EPTB-DM-HEP followed by EPTB-DM-HIV. However, higher proportions of default were seen among patients with EPTB-DM (Fig. 2).

### Risk factors of EPTB

With EPTB as the case group and PTB as the control group, we assessed the associations between the variables and having EPTB based on logistic regression model. Based on the result of the adjusted odd ratio (AOR), females appeared to have a higher risk for having EPTB (1.524 [CI: 1.311–1.746]; *p* <  0.001) than males (Table 5). Malays (1.251 [1.056–1.482]; *p =* 0.010), Indians (1.450 [1.142–1.842]; *p =* 0.002), urban residents (1.272 [1.109–1.459]; *p =* 0.001), patients with no formal education (1.361 [1.018–1.820]; *p =* 0.037), those with married (1.199 [1.038–1.384]; *p =* 0.014) and with unknown marital status (2.757 [1.611–4.717]; *p* <  0.001) had significantly higher odds for having EPTB. Among the co-morbid conditions, patients with EPTB-HIV (3.215 [2.347–4.405]; *p* <  0.001), EPTB-DM-HIV (4.361 [1.657–11.474]; *p =* 0.003) and EPTB-HIV-HEP (4.083 [2.785–5.987]; *p* <  0.001) found to have increased risk of EPTB.

**Table 5:** Logistic regression models to determine independent risk factors for having EPTB

Variables	Univariate analysis	*P*-value	Multivariate analysis	*P*-value
	COR (95% CI)		AOR (95% CI)	
Gender				
Male	0.75 (0.66 to 0.86)	< 0.001	0.65 (0.57 to 0.75)	< 0.001
Female	1.31 (1.16 to 1.49)		1.52 (1.31 to 1.74)	
Age (years)				
≤ 15	1.115 (0.78 to 1.58)	0.541		
16–25	1.11 (0.95 to 1.31)	0.170		
26–35	0.90 (0.77 to 1.05)	0.198	-----------------------	-------
36–45	0.96 (0.82 to 1.12)	0.604		
46–55	1.02 (0.87 to 1.20)	0.741		
56–65	0.94 (0.79 to 1.13)	0.566		
≥ 66	1.06 (0.87 to 1.28)	0.546		
Ethnicity				
Malay	1.33 (1.16 to 1.51)	< 0.001	1.25 (1.05 to 1.48)	0.010
Chinese	1.02 (0.89 to 1.17)	0.740	---------------------	-------
Indian	1.58 (1.28 to 1.94)	< 0.001	1.45 (1.14 to 1.84)	0.002
Immigrants Indonesian	0.72 (0.55 to 0.94)	0.018	0.76 (0.56 to 1.02)	0.069
Immigrants Philippines	0.65 (0.47 to 0.89)	0.008	0.77 (0.55 to 1.09)	0.144
Sarawakian	0.61 (0.48 to 0.77)	< 0.001	0.76 (0.58 to 0.99)	0.046
Sabahian	0.80 (0.68 to 0.94)	0.009	0.91 (0.74 to 1.12)	0.388
Others	1.63 (1.08 to 2.46)	0.019	1.51 (0.97 to 2.35)	0.064
Residence				
Urban	1.17 (1.03 to 1.33)	0.015	1.27 (1.10 to 1.45)	0.001
Rural	0.85 (0.75 to 0.97)		0.78 (0.68 to 0.90)	
Patient category				
Unknown	0.30 (0.20 to 0.46)	< 0.001	0.21 (0.12 to 0.35)	< 0.001
New	1.92 (1.53 to 2.40)	< 0.001	1.57 (1.20 to 2.05)	0.001
Relapse	0.73 (0.56 to 0.94)	0.017	4.65 (2.80 to 7.73)	< 0.001
Co-morbidity				
Only TB	0.73 (0.65 to 0.83)	< 0.001	0.89 (0.68 to 1.16)	0.406
TB-DM	0.66 (0.56 to 0.78)	< 0.001	0.67 (0.50 to 0.91)	0.011
TB-HIV	2.35 (1.97 to 2.80)	< 0.001	3.21 (2.34 to 4.40)	< 0.001
TB-Hep	0.99 (0.75 to 1.31)	0.990	---------------------	-------
TB-DM-HIV	3.33 (1.34 to 8.27)	0.009	4.36 (1.65 to 11.47)	0.003
TB-DM-Hep	1.30 (0.68 to 2.50)	0.419	----------------------	-------
TB-HIV-Hep	3.01 (2.30 to 3.95)	< 0.001	4.08 (2.78 to 5.98)	< 0.001
Smoking habit				
Ex-smoker	1.54 (1.28 to 1.84)	< 0.001	1.53 (1.26 to 1.87)	< 0.001
No	0.96 (0.85 to 1.09)	0.599	----------------------	-------
Yes	0.85 (0.75 to 0.97)	0.017	0.78 (0.67 to 0.91)	0.001
Drinking Habit				
Unknown	0.73 (0.40 to 1.33)	0.314		
No	0.95 (0.78 to 1.15)	0.623	-----------------------	-------
Yes	1.10 (0.89 to 1.35)	0.356		
IVDU^a^				
Unknown	1.60 (1.01 to 2.53)	0.044	1.16 (0.67 to 2.01)	0.578
No	0.75 (0.61 to 0.94)	0.012	0.93 (0.71 to 1.22)	0.624
Yes	1.24 (0.98 to 1.58)	0.071	----------------------	--------
Marital status				
Unknown	2.68 (1.63 to 4.42)	< 0.001	2.75 (1.61 to 4.71)	< 0.001
Married	1.18 (1.03 to 1.34)	0.013	1.19 (1.03 to 1.38)	0.014
Unmarried	0.80 (0.70 to 0.91)	0.001	0.36 (0.21 to 0.62)	< 0.001
Education				
Unknown	0.87 (0.76 to 0.99)	0.041	0.95 (0.80 to 1.13)	0.603
Primary	0.90 (0.73 to 1.10)	0.319	----------------------	--------
Secondary	1.23 (0.97 to 1.57)	0.081	----------------------	--------
College	0.89 (0.64 to 1.24)	0.499	----------------------	--------
University	1.10 (0.52 to 2.34)	0.790	----------------------	--------
Diploma	1.43 (0.88 to 2.33)	0.146	----------------------	--------
No formal education	1.55 (1.21 to 2.00)	0.001	1.36 (1.01 to 1.82)	0.037
Employment status				
Unknown	1.17 (0.99 to 1.39)	0.057		
Employed	1.06 (0.92 to 1.21)	0.377	----------------------	--------
Unemployed	0.87 (0.77 to 0.99)	0.033		

*TB-DM* Co-infection of TB and Diabetes Mellitus, *TB-HIV* Co-infection of TB and HIV, *TB-Hep* Co-infection of TB and Hepatitis, *TB-DM-HIV* Co-infection of TB with Diabetes Mellitus and HIV, *TB-DM-Hep* Co-infection of TB with Diabetes Mellitus and Hepatitis, *TB-HIV-Hep* Co-infection of TB with HIV and Hepatitis

^a^Intravenous Drug Users

## Discussion

To the best of our knowledge, this is the first study in Malaysia to describe the epidemiological, clinical characteristics and treatment outcomes among patients with EPTB and its co-morbidities. Patients with EPTB constituted 13.1% of all notifications, with some parts of the country showing higher prevalence than others. There were important variations in the proportion of EPTB patients in the different states of the country; and this could be related to the implication of medical doctors in the diagnosis of EPTB. The proportion of patients diagnosed with EPTB in the present study was lower than that reported from other parts of world [14–16]. Predominant sites of EPTB were lymph node followed by pleural effusion. The higher prevalence of lymph node and pleural effusion has previously been reported in Malaysia and other global regions [6, 15, 17, 18]. The other rare forms included TB of the eye, ear, breast, neck, skin and spondylitis. Beside this, there were 18 cases who had EPTB at more than one site.

Frequency of different sites of EPTB varied among co-morbidities. Lymph node and pleural effusion were observed at higher proportion, followed by miliary and meningitis TB. Association between HIV and sites of EPTB has been determined more than a decade ago [19] however; the data is limited or almost absent for DM and HEP. Consistent with previous studies [20, 21] we found advanced HIV strongly correlated with the occurrence of EPTB. These findings are in contrast to [22] but are in agreement with [23]. Furthermore, severe immunosuppression like low CD4+ lymphocyte cell counts and advanced HIV infection, increases the risk of having EPTB as opposed to PTB alone [24, 25]. Moreover, on comparing CD4+ lymphocyte cell counts with smoking, CD4+ lymphocyte cell counts < 100 was significantly recorded for smokers (*p* = < 0.05). This is the first study in identifying smoking association with CD4+ lymphocyte cell counts < 100 among EPTB-HIV. The previous study by Feldman and companions suggested lower CD4+ lymphocyte cell counts in HIV patients with smoking habit [26].

A significant association was observed between co-morbidities and age, gender, ethnicity, patient category, education and marital and employment status. Proportions of co-morbidities were greater in males, unmarried and unemployed patients comparative to their counterparts. The results show that the risk of developing co-morbidities remained higher at the age of 26 years and older. Of total 1222 patients in the present study, 525 were recorded for different co-morbidities with EPTB-HIV and EPTB-DM being the most common. Moreover, 11.4% of patients had EPTB-HEP and 15% cases were seen with EPTB-HIV-HEP co-infection. During last decade, one case-control study in US demonstrated association of hepatitis C infection with TB disease [27]. Later on, it was confirmed by further studies showing that hepatitis C infection and TB share the same high risks population [28–30]. Very recently study conducted in Taiwan has reported that hepatitis C infection intensifies the risk of developing TB [31]. The mechanism behind this finding remains unclear. Future studies in this perspective are needed.

Treatment success rate in our study was 67.6% (826/1222). On multivariate analysis, age group 56–65 years, relapse cases, EPTB-DM, patients with no formal and secondary level of education were recorded as statistically positive significant risk factors for unsuccessful treatment outcomes. Treatment success rate among patients on HAART was 79.9% which is far better than that mentioned in a study conducted in Kelantan, north-east Malaysia [32]. On the other side, EPTB-DM patients had higher odds for unsuccessful treatment outcomes. Poor outcomes in patients with DM-TB could be due to immune deficiency triggered by diabetes [33]. Increased deaths were observed in meningitis, miliary TB, EPTB-DM-HEP and EPTB-DM-HIV. Meningeal TB is particularly challenging to diagnose, since cerebrospinal fluid is commonly smear and culture negative. Meningitis and a CD4+ lymphocyte cell counts < 200 have been reported as risk factor for deaths among EPTB patients by [25]. Meningitis, disseminated disease, patients with EPTB-HIV and EPTB-DM also have been reported as risk factors of poor TB outcomes, including increased mortality in other studies [22, 33].

The finding of females, Malays, Indians, urban residents, patients with EPTB-HIV, EPTB-DM-HIV and EPTB-HIV-HEP as independent predictors for having EPTB at the study sites is consistent with studies from other countries [3, 34–37]. The differences in the proportion of EPTB by ethnicity are notable. Malay and Indian patients were generally far more likely to present with EPTB than others. Differences in the likelihood of EPTB for racial differences have been observed in various studies [16, 38]. The mechanism of a racial difference in infectiousness by MTB is the result of a complex interaction between the environmental, immunologic and genetic factors [38]. However, more studies among larger number of patients are needed to further ratify these results. Weak immune system among DM patients could led them to get infections, including TB [39]. Patients with DM are identified as risk factors for PTB in numerous studies [39, 40] but data is scarce among EPTB patients. One of the remarkable finding of our study therefore includes patients with DM at greater risk of EPTB that is in line with the study conducted at Georgia [33].

## Limitations

This study has some limitations for its retrospective nature. We could not assess whether patients who completed treatment increased their weight. Beside this, documentation of diabetes, hepatitis and HIV was likely to be incomplete. In addition, effect of TB treatment on CD4+ lymphocyte cell counts was not studied.

## Conclusions

With continuous growing trend, EPTB is a grave concern to public health in Malaysia for mainly affecting nationals. High prevalence of EPTB-DM, EPTB-HIV and EPTB-HEP as well as their further compound co-morbidities among EPTB in the present study signifies the fact that these patients are at high risk of developing EPTB. Active screening measures for patients with co-morbidities are therefore recommended in patients with EPTB which could improve the diagnosis and early management of co-morbidities complications. This strategy together with educating patients can further increase the treatment success rate.

## Data Availability

The datasets used and/or analyzed during the current study are available from the corresponding author on reasonable request.

## Abbreviations

AOR: Adjusted odds ratio
CI: Confidence intervals
COR: Crude odds ratio
DM: Diabetes Mellitus
EPTB: Extra-pulmonary tuberculosis
HAART: Highly Active Antiretroviral Therapy
MTB: *Mycobacterium tuberculosis*
NIMR: National Institute for Medical Research, London
NTP: National TB Control Program
PCR: Polymerase Chain Reaction
PTB: Pulmonary tuberculosis
TB: Tuberculosis
WHO: World Health Organization

## References

[1] World Health Organization. WHO. Global Tuberculosis Report 2017. Geneva. WHO/HTM/TB/2017.23.

[2] Guler SA, Bozkus F, Inci MF, Kokoglu OF, Ucmak H, Ozden S, Yuksel M (2015). Evaluation of pulmonary and extrapulmonary tuberculosis in immunocompetent adults: a retrospective case series analysis. Med Princ Pract.

[3] Yang Z, Kong Y, Wilson F, Foxman B, Fowler AH, Marrs CF, Cave MD, Bates JH (2004). Identification of risk factors for extrapulmonary tuberculosis. Clin Infect Dis.

[4] Sunnetcioglu A, Sunnetcioglu M, Binici I, Baran AI, Karahocagil MK, Saydan MR (2015). Comparative analysis of pulmonary and extrapulmonary tuberculosis of 411 cases. Ann Clin Microbiol Antimicrob.

[5] Ramirez-Lapausa M, Menendez-Saldana A, Noguerado-Asensio A (2015). Extrapulmonary tuberculosis: an overview. Rev Esp Sanid Penit.

[6] Iyawoo K (2004). Tuberculosis in Malaysia: problems and prospect of treatment and control. Tuberculosis.

[7] Council MA (2018). Overview of the HIV & AIDS epidemic in Malaysia.

[8] Jeon CY, Murray MB (2008). Diabetes mellitus increases the risk of active tuberculosis: a systematic review of 13 observational studies. PLoS Med.

[9] Baker MA, Lin H-H, Chang H-Y, Murray MB (2012). The risk of tuberculosis disease among persons with diabetes mellitus: a prospective cohort study. Clin Infect Dis.

[10] Sulaiman SAS, Khan AH, Ahmad N, Iqubal MS, Muttalif AR, Hassali MA (2013). Impact of diabetes mellitus on treatment outcomes of tuberculosis patients in tertiary care setup. Am J Med Sci.

[11] WHO. Global Tuberculosis Report. Geneva; 2013. WHO/HTM/TB/2013.11

[12] MOH (2012). Clinical practice guidelines.

[13] WHO. Treatment of Tuberculosis Guidelines. Geneva; 2009. WHO/HTM/TB/2009.420

[14] Rock RB, Sutherland WM, Baker C, Williams DN (2006). Extrapulmonary tuberculosis among Somalis in Minnesota. Emerg Infect Dis.

[15] te Beek LAM, van der Werf MJ, Richter C, Borgdorff MW (2006). Extrapulmonary tuberculosis by nationality, the Netherlands, 1993–2001. Emerg Infect Dis.

[16] Forssbohm M, Zwahlen M, Loddenkemper R, Rieder HL (2008). Demographic characteristics of patients with extrapulmonary tuberculosis in Germany. Eur Respir J.

[17] Peto HM, Pratt RH, Harrington TA, LoBue PA, Armstrong LR (2009). Epidemiology of Extrapulmonary tuberculosis in the United States, 1993–2006. Clin Infect Dis.

[18] Nissapatorn V, Kuppusamy I, Rohela M, Anuar AK, Fong M (2004). Extrapulmonary tuberculosis in peninsular Malaysia: retrospective study of 195 cases. Southeast Asian J Trop Med Public Health.

[19] Yechoor VK, Shandera WX, Rodriguez P, Cate TR (1996). Tuberculous meningitis among adults with and without HIV infection: experience in an urban public hospital. Arch Intern Med.

[20] Namme L, Marie-Solange D, Hugo Bertrand M, Elvis T, Achu J, Christopher K (2013). Extrapulmonary tuberculosis and HIV coinfection in patients treated for tuberculosis at the Douala general hospital in Cameroon. Ann Trop Med Public Health.

[21] Kipp AM, Stout JE, Hamilton CD, Van Rie A (2008). Extrapulmonary tuberculosis, human immunodeficiency virus, and foreign birth in North Carolina, 1993–2006. BMC Public Health.

[22] Kourbatova EV, Leonard MK, Romero J, Kraft C, del Rio C, Blumberg HM (2006). Risk factors for mortality among patients with extrapulmonary tuberculosis at an academic inner-city hospital in the US. Eur J Epidemiol.

[23] Annie L. Tuberculosis and HIV. In: HIV InSite. San Francisco: University of California San Francisco; 2013. http://hivinsite.ucsf.edu/InSite?page=kb-05-01-06. Accessed Jan 2013.

[24] Jones BE, Young SM, Antoniskis D, Davidson PT, Kramer F, Barnes PF (1993). Relationship of the manifestations of tuberculosis to CD4 cell counts in patients with human immunodeficiency virus infection. Am J Respir Crit Care Med.

[25] Kingkaew N, Sangtong B, Amnuaiphon W, Jongpaibulpatana J, Mankatittham W, Akksilp S (2009). HIV-associated extrapulmonary tuberculosis in Thailand: epidemiology and risk factors for death. Int J Infect Dis.

[26] Feldman JG, Minkoff H, Schneider MF, Gange SJ, Cohen M, Watts DH, Gandhi M, Mocharnuk RS, Anastos K (2006). Association of cigarette smoking with HIV prognosis among women in the HAART era: a report from the women’s interagency HIV study. Am J Public Health.

[27] El-Serag HB, Anand B, Richardson P, Rabeneck L (2003). Association between hepatitis C infection and other infectious diseases: a case for targeted screening?. Am J Gastroenterol.

[28] Reis N, Lopes C, Teles SA, Matos M, Carneiro M, Marinho T (2011). Hepatitis C virus infection in patients with tuberculosis in Central Brazil. Int J Tuberc Lung Dis.

[29] Beijer U, Wolf A, Fazel S (2012). Prevalence of tuberculosis, hepatitis C virus, and HIV in homeless people: a systematic review and meta-analysis. Lancet Infect Dis.

[30] Awofeso N (2010). Prisons as social determinants of hepatitis C virus and tuberculosis infections. Public Health Rep.

[31] Wu P-H, Lin Y-T, Hsieh K-P, Chuang H-Y, Sheu C-C (2015). Hepatitis C virus infection is associated with an increased risk of active tuberculosis disease: a nationwide population-based study. Medicine..

[32] Jalal TMT, Abdullah S, Wahab FA, Dir S, Naing NN (2017). Prevalence and factors associated with tuberculosis treatment success among TB/HIV co-infection in North-East Malaysia. Malays J Med Sci.

[33] Magee M, Foote M, Ray S, Gandhi N, Kempker R (2016). Diabetes mellitus and extrapulmonary tuberculosis: site distribution and risk of mortality. Epidemiol Infect.

[34] Ade S, Harries AD, Trébucq A, Ade G, Agodokpessi G, Adjonou C, Azon S, Anagonou S (2014). National profile and treatment outcomes of patients with extrapulmonary tuberculosis in Bénin. PLoS One.

[35] Wang X, Yang Z, Fu Y, Zhang G, Wang X, Zhang Y, Wang X (2014). Insight to the epidemiology and risk factors of extrapulmonary tuberculosis in Tianjin, China during 2006-2011. PLoS One.

[36] Gomes Nathália Mota de Faria, Bastos Meire Cardoso da Mota, Marins Renata Magliano, Barbosa Aline Alves, Soares Luiz Clóvis Parente, de Abreu Annelise Maria de Oliveira Wilken, Souto Filho João Tadeu Damian (2015). Differences between Risk Factors Associated with Tuberculosis Treatment Abandonment and Mortality. Pulmonary Medicine.

[37] Sanches I, Carvalho A, Duarte R (2015). Who are the patients with extrapulmonary tuberculosis?. Rev Port Pneumol (English Edition).

[38] Fares A (2012). Racial differences in susceptibility to infection by mycobacterium tuberculosis. Ann Trop Med Public Health.

[39] Agarwal AK, Ginisha G, Preeti G, Dwivedi S, Swamai P (2016). The association between diabetes and tuberculosis may be the next challenge for global tuberculosis control worldwide. Indian J Endocrinol Metab.

[40] Leung CC, Lam TH, Chan WM, Yew WW, Ho KS, Leung GM (2008). Diabetic control and risk of tuberculosis: a cohort study. Am J Epidemiol.

